# Strangles in South Africa

**Published:** 1896-03

**Authors:** 


Strangles in South Africa. From an article by Dr. Sander on "South African Epidemics" in the January *Archiv für wissenschaftliche und praktische Thierheilkunde*.

In regard to the name, it is to be noticed that this disease is often called the "new sickness" in South Africa. However, in Cape Colony the same name is often applied to what we call glanders, so that ambiguity often arises in articles dealing with diseases in South Africa.

The time for the appearance of the disease (strangles) is in accordance with what I have observed myself and heard from the settlers, the summer months, which naturally correspond to our winter months on the calendar. For the district under consideration (Windhoek), as well as for the greater part of South Africa, the summer and the rainy season are identical. For this reason the settlers always designate the rainy season as the time for the appearance of the disease. However, this was only true of one of the two cases which I had the opportunity to observe. The other case occurred in January, 1894, long before the rainy season, which was very late that year, had set in.

The disease is not contagious to a degree worthy of mention, for both the horses under my observation stood together with several others in the same stall, respectively kraal, and went together with these and others to the same pasture without communicating the disease to any of the others. This argues against a high degree of infectiousness. However, that the disease is contagious to a small degree is not to be denied, because both horses were taken sick at the same place and in the same pasture, while at other places in the district the disease, as far as I know, did not appear in 1894. The second horse was taken sick at the end of March or the beginning of April, after the disease in the first had almost run its course.

The symptoms in both cases consisted of swellings in the region of the cheeks (jowls), corresponding to the position of the antrum Highmori. I found here a hard, semi-spherical swelling about the size of a goose-egg in which fluctuation was not perceptible. Examination from the cavity of the mouth showed that the swelling was not confined to the skin, as the impossibility to move it aside on the parts underlying had already made probable, but that it had spread to the underlying structures. On account of the great hardness of the tumor and my inability to separate it from the bone or push it aside upon the same, I received the impression that I might have to deal here with a catarrhal condition of the Highmore cavity, together with obstruction of the channels leading to the nose. A considerable degree of catarrh in the mucous membrane of the nostrils and of the (connecting skin) of the eyelids seemed to add support to this view. In spite of this, however, I believe that it was merely a hard infiltration of the soft parts.

In the further course of the disease pus was formed and broke through ; in the first case, on the outside ; in the second, on the inside into the cavity of the mouth. The eruption of pus on the inside is said always to take place into the cavity of the mouth, and not, for example, into the cavities of the nose. At least such is the information that I have received. Besides, the tumor, in other cases which have shown in other respects the same general symptoms, was found on the under jawbone, corresponding to the catarrh in the mucous membrane of the respiratory channels. A disturbance of the general condition was noticeable in both cases, which consisted in inertness, ravenous appetite, languid look out of the eye, and in moderate coughing.

Sickness set in pretty suddenly, then took a chronic course and disappeared after several weeks. As soon as the pus had broken out the general symptoms disappeared, and the horses were again put to work.

The treatment consists in an artificial opening of the swelling if it does not reach an eruption in the natural course. If the pus is not gotten rid of, or it is impossible to open the tumor because it lies too deeply imbedded, the animals are said to die.

